# Viability Assessment During Normothermic Machine Liver Perfusion: A Literature Review

**DOI:** 10.1111/liv.16244

**Published:** 2025-01-17

**Authors:** Heithem Jeddou, Stylianos Tzedakis, Mohamed Ali Chaouch, Laurent Sulpice, Michel Samson, Karim Boudjema

**Affiliations:** ^1^ Department of Hepatobiliary and Digestive Surgery University Hospital, Rennes 1 University Rennes France; ^2^ Inserm, EHESP, Irset (Institut de recherche en santé, environnement et travail)‐UMR_S 1085, Université de Rennes Rennes France; ^3^ Department of Hepato‐Biliary, Digestive and Endocrine Surgery Cochin Hospital, APHP Paris France; ^4^ Université Paris Cité Paris France; ^5^ Department of Visceral and Digestive Surgery Monastir University Hospital Monastir Tunisia; ^6^ INSERM OSS U1242, University Hospital, Rennes 1 University Rennes France

**Keywords:** liver transplantation, normothermic machine perfusion, viability assessment

## Abstract

Background and Objective

The discrepancy between donor organ availability and demand leads to a significant waiting‐list dropout rate and mortality. Although quantitative tools such as the Donor Risk Index (DRI) help assess organ suitability, many potentially viable organs are still discarded due to the lack of universally accepted markers to predict post‐transplant outcomes. Normothermic machine perfusion (NMP) offers a platform to assess viability before transplantation. Thus, livers considered unsuitable for transplantation based on the DRI can be evaluated and potentially transplanted. During NMP, various viability criteria have been proposed. These criteria are neither homogeneous nor consensual. In this review, we aimed to describe the viability criteria during NMP and evaluate their ability to predict hepatic graft function following transplantation. We conducted a PubMed search using the terms ‘liver transplantation’, ‘normothermic machine perfusion’ and ‘assessment’, including only English publications up to February 2024. Viability assessment during NMP includes multiple hepatocellular and cholangiocellular criteria. Lactate clearance and bile production are commonly used indicators, but their ability to predict post‐transplant outcomes varies significantly. The predictive value of cholangiocellular criteria such as bile pH, bicarbonate and glucose levels remains under investigation. Novel markers, such as microRNAs and proteomic profiles, offer the potential to enhance graft evaluation accuracy and provide insights into the molecular mechanisms underlying liver viability. Combining perfusion parameters with biomarkers may improve the prediction of long‐term graft survival. Future research should focus on standardising viability assessment protocols and exploring real‐time biomarker evaluations, which could enhance transplantation outcomes and expand the donor pool.

**Summary**

- This study examines how advanced techniques help assess the suitability of donated livers for transplantation.
- Doctors can evaluate the organ's health before surgery by using a specialised machine to keep the liver functioning outside the body, potentially saving lives by utilising organs that might otherwise have been discarded.

**Abbreviations**
ALT: alanine aminotransferase
AST: aspartate aminotransferase
ATP: adenosine triphosphate
COR: controlled oxygenated rewarming
DBD‐ECD: extended criteria brain‐dead donors
DCD: donation after circulatory death
DHOPE: dual hypothermic oxygenated machine perfusion
DRI: donor risk index
EAD: early allograft dysfunction
IC: ischaemic cholangiopathy
IL‐6: interleukin 6
IL‐10: interleukin 10
IRI: ischaemia–reperfusion injury
LDH: lactate dehydrogenase
LT: liver transplantation
NAS: non‐anastomotic biliary strictures
NMP: normothermic machine perfusion
PNF: primary nonfunction
PRBC: packed red blood cells
SCS: static cold storage
SLAC: standardised lactic acid clearance

## 1 Introduction

Liver transplantation (LT) is a highly effective treatment for end‐stage liver disease and early‐stage primary liver cancer [1]. Over the last half century, the evolution of transplantation techniques has led to significant improvements, with an overall 5‐year survival of 70%–80% [2]. However, the persistent gap between the availability and demand of donor organs has resulted in an ever‐increasing dropout and mortality rate in transplant waitlists. In 2023, in France, 18% of patients on the waitlist either died or were removed due to worsening of their condition [2].

To address the organ shortage, donor acceptance criteria have been expanded to extended criteria brain‐dead donors (DBD‐ECD), which include characteristics such as advanced age or hepatic steatosis, as well as donation after circulatory death (DCD) [3]. Unfortunately, DBD‐ECD and DCD livers are more vulnerable to ischaemia–reperfusion injury (IRI) during liver transplantation. Ischaemic injury results from oxygen depletion, glycogen loss and reduced ATP levels, which impair cellular metabolism. Reperfusion further exacerbates this damage, leading to microvascular disturbances, thrombosis and biliary strictures [4].

Although all types of ischaemia share common mechanisms, cold ischaemia mainly causes damage to the sinusoidal lining cells and disrupts the microcirculation, while warm ischaemia primarily leads to hepatocellular injury mediated by cytotoxic molecules released by Kupffer cells [4]. During liver transplantation, IRI also impacts the peribiliary plexus, triggering endothelial cell activation, which leads to microvascular thrombosis, microcirculatory disturbances and additional ischaemia [4, 5].

These factors contribute to biliary strictures, cholangiocyte apoptosis, necrosis and cholangitis. Cholangiocytes are particularly sensitive to ischaemic injury, recovering ATP more slowly than hepatocytes, making them vulnerable during the reperfusion phase [4, 5]. Consequently, DBD‐ECD and DCD livers display a higher incidence of early allograft dysfunction (EAD), primary nonfunction (PNF) and post‐transplant biliary complications such as ischaemic cholangiopathy, which ultimately reduce graft and recipient survival [6, 7, 8, 9].

The suitability of donor livers is traditionally based on donor history, age, weight, biological parameters of liver function, radiologic features and the macroscopic aspect of the graft at the time of procurement. However, the predictive value of these parameters to detect grafts unsuitable for transplantation is low, especially in the group of grafts obtained from DBD‐ECD and DCD [10]. Quantitative tools such as the Donor Risk Index (DRI) have been developed to aid in assessing organ viability [11, 12]. Despite the use of these tools, many potentially viable organs are still discarded, underscoring the continued dependence on subjective assessment and clinical judgement in the absence of universally accepted viability markers. In 2023, in France, 310 (21%) proposed livers were rejected [2]. An effective and objective means of pretransplant viability assessment is necessary to allow for greater use of marginal livers.

The current standard of donor liver preservation is based on static cold storage (SCS), where organs are flushed and cooled with specific chilled preservation solutions, transported and stored in an ice box in preservation solution until transplantation. While SCS is the most common preservation method, it does not allow assessment of liver viability after cold ischaemia [13].

In response, ex situ machine perfusion has gained popularity for evaluating grafts before implantation [14, 15, 16, 17, 18]. Hypothermic perfusion is less effective for assessing liver viability, as hepatic metabolism remains significantly reduced under hypothermic conditions, and bile production—a crucial liver function involving hepatocytes and cholangiocytes—does not occur during this process, making it difficult to evaluate liver function and viability [19]. However, during normothermic machine perfusion (NMP), the liver receives oxygen, nutrients and medications at physiological temperatures and pressures, maintaining physiological conditions that support homeostasis and normal metabolic activity. This approach not only allows preservation of the liver but also functional assessment of the graft, providing the opportunity to assess key processes that can be used to determine organ viability [20, 21, 22]. Therefore, livers initially deemed unsuitable for transplantation can be evaluated on NMP, and those meeting viability criteria may be considered for transplantation, thereby increasing the pool of suitable liver grafts.

In this study, we present an overview of the criteria, indicators and methods used to evaluate liver viability for transplantation, which are available in the literature. We aim to describe the viability criteria during NMP and evaluate their ability to predict the hepatic graft function following transplantation. Additionally, we examine the current challenges in liver transplantation and provide future perspectives.

## 2 Methods

This narrative review was conducted through an extensive literature search of the PubMed database using the terms ‘liver transplantation’, ‘normothermic machine perfusion’ and ‘assessment’. Only English publications up to February 2024 were included. Criteria for assessing hepatocellular viability, such as lactate clearance, bile production, glucose metabolism, perfusate pH, hemodynamic stability, liver transaminase levels and cholangiocellular viability, were analysed. The discussion includes the predictive value of these parameters as well as emerging biomarkers.

### 2.1 Criteria Used for Assessment of Viability During NMP

The functional assessment of the liver graft covers two compartments of the liver: hepatocytes, responsible for initial liver function and cholangiocytes, which are predominantly involved in late biliary strictures (Table 1). Hepatocellular viability, focussing on the metabolic functionality of the liver parenchyma, is assessed using criteria such as lactate clearance and bile production [25]. Biliary viability, which focusses on the functional capacity of cholangiocytes, is assessed by the specific biochemical composition of bile [17, 25, 26, 27].

**TABLE 1:** Hepatocellular and cholangiocellular viability criteria during NMP.

	Hepatocellular criteria	Cholangiocellular criteria
Birmingham criteria [23]	Viability is assessed within 4 h of perfusion: Perfusate lactate level ≤ 2.5 mmol/L, evidence of bile production, perfusate pH ≥ 7.30, metabolism of glucose, stable arterial flow ≥ 150 mL/min and portal flow ≥ 500 mL/min, homogeneous graft perfusion with soft consistency of the parenchyma	
Cambridge criteria [13]	Viability is assessed within 2 h of perfusion: Perfusate pH > 7.20 without more than 30 mL bicarbonate supplementation, falling perfusate glucose beyond 2 h or perfusate glucose < 10 mmol/L, perfustae alanine aminotransferase (ALT) < 6000 IU/L, peak lactate fall ≥ 4.4 mmol/L/kg/h	Bile pH > 7.5 Bile glucose concentration ≤ 3 mmol/L or ≥ 10 mmol but< perfusate glucose
Groningen criteria [24]	Viability is assessed within 2.5 h of perfusion: Perfusate lactate level < 1.7 mmol/L, perfustae pH within the range 7.35 to 7.45, cumulative bile production ≥ 10 mL	Biliary pH > 7.45, Δ pH > 0.10, Δ bicarbonates > 5 mmol/L, Δ Glucose < −5 mmol/L

### 2.2 Hepatocellular Viability Criteria

#### 2.2.1 Lactate Clearance

**FIGURE 1:**
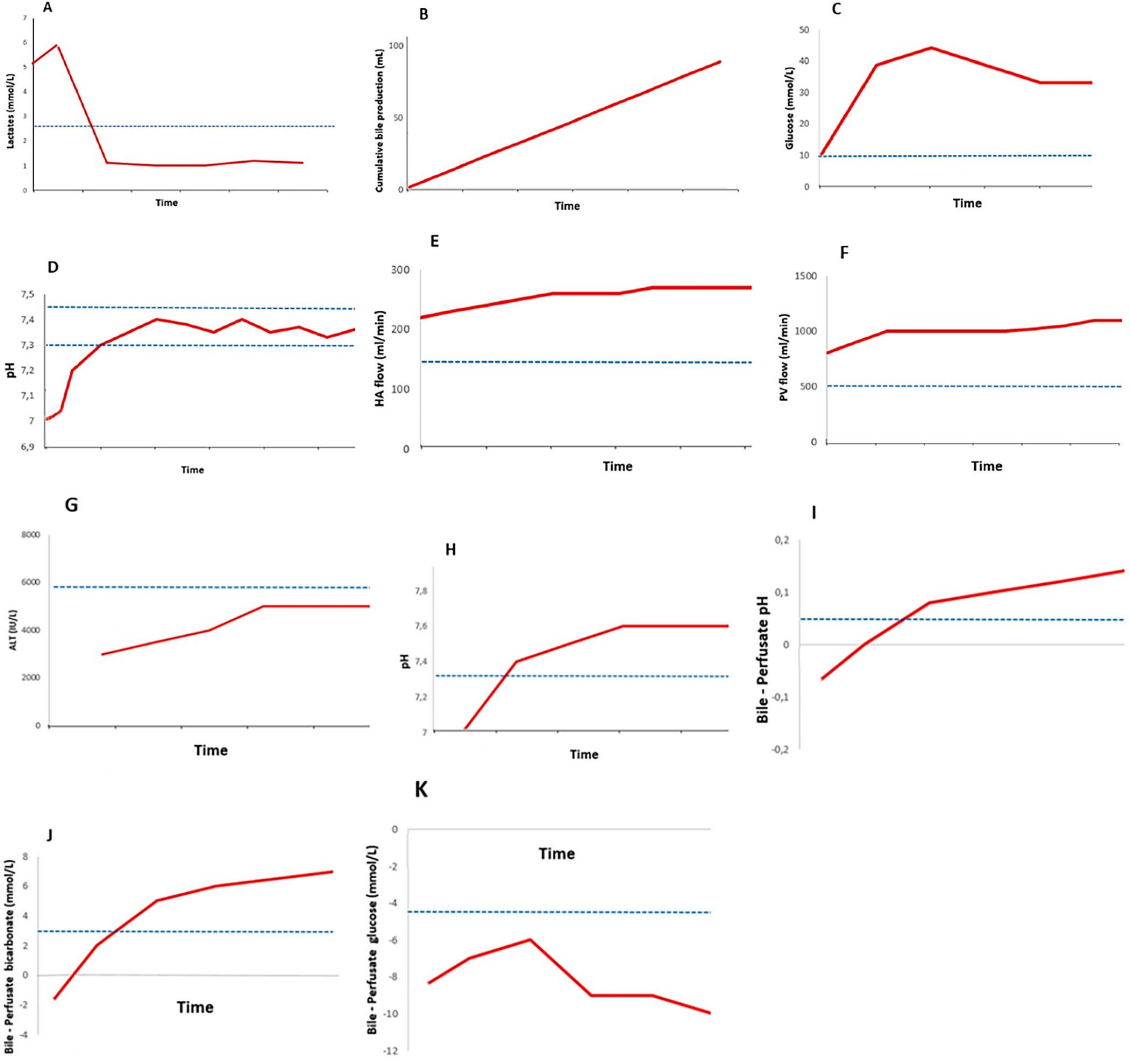
Perfusate and bile characteristics of viable livers during NMP. The dotted lines represent cutoff values used as viability criteria in clinical studies. Figures are based on the studies listed in Table 1, adapted from Brüggenwirth [18] et al. and Watson et al. [29]. (A) perfusate lactate, (B) bile production, (C) perfusate glucose, (D) perfusate pH, (E) HA flow, (F) PV flow, (G) transaminases in perfusate, (H) bile pH, (I) Δ pH, (J) Δ bicarbonates, (K) Δ Glucose.

Lactate, a by‐product of anaerobic glycolysis, is a traditional marker of tissue hypoperfusion and hypoxia. Under normal conditions, the liver plays a key role in lactate processing by converting it back to pyruvate via lactate dehydrogenase. Thus, lactate levels represent a dynamic biomarker for monitoring liver graft function [23]. During NMP, the following three lactate phases are generally observed in a functioning liver: an initial lactate peak up to 1 h after NMP initiation, followed by a rapid drop within 2 h and then a steady low state for the remaining perfusion time [28] (Figure 1A).

Lactate clearance is the most widely used parameter for assessing viability during NMP (Table 2). The Birmingham group established viability criteria based on preclinical studies of discarded livers, showing that perfusate lactate clearance, combined with bile production and stable blood flow, predicts graft viability [38]. In August 2014, they performed the first‐in‐human transplant of such a liver graft [39]. In 2016, the same group assessed the viability of six declined liver grafts (four DCD and two *DBD‐ECD*) using NMP. The perfusion fluid consisted of packed red blood cells (PRBC), supplemented with human albumin solution. Two devices were used: the Liver Assist (Organ Assist, Groningen, the Netherlands) for five livers, and the OrganOx Metra (OrganOx, Oxford, UK) for one liver. Organ viability was assessed within 3 h of perfusion. A liver was considered viable when the perfusate lactate level was less than 2.5 mmol/L or the liver produced bile, in combination with at least two of the following criteria: (i) perfusate pH greater than 7.30, (ii) stable arterial flow more than 150 mL/min and portal venous flow more than 500 mL/min and (iii) homogeneous graft perfusion with soft consistency of the parenchyma (Table 1). All but one liver met the defined criteria for viability and were subsequently transplanted. After a median follow‐up of 7 months, all recipients were alive with normalised liver function tests [10].

**TABLE 2:** Viability criteria applied in clinical studies for assessing liver viability during NMP before liver transplantation.

Author & Year	Country	Number of transplanted livers/ perfused livers (Rescue rate)	Type of transplanted Livers	Indication of perfusion	Device used	Composition of the perfusate	Duration of storage of transplanted livers	Viability Criteria	Outcomes and follow up
Mergental et al. [21]	UK	22/31 (71%)	12 DBD‐ECD 10 DCD	Viability testing of initially discarded livers	OrganOx metra device	Packed red blood cells	Median CIT: 464 min Median WIT for DCD: 21 min Median perfusion time: 587 min	Within 4 h of NMP: Lactate < 2.5 mmol/L and at least 2 of the following criteria: Evidence of bile production Perfusate pH > 7.30 Metabolism of glucose HA flow > 150 mL/min and PV flow > 500 mL/min Homogenous graft perfusion	No PNF 4 NAS required retransplantation Follow up: 5 years
Olumba et al. [30]	United states	16/22 (72.7%)	12 DBD‐ECD 10 DCD	Viability testing of initially discarded livers	OrganOx metra device	Packed red blood cells	Median CIT: 385 min Median WIT for DCD: 24.5 min Median perfusion time: 561 min	Within 2 h of NMP: Perfusate lactate ≤ 2.2 mmol/L Hepatic arterial flow ≥ 0.1 L/min, and portal flow ≥ 0.5 L/min And at least two of the following criteria: Metabolism of glucose Perfusate pH ≥ 7.25 with bicarbonate use ≤ 70 mL Bile production Perfusate ALT ≤ 7000 U/L and AST ≤ 10 000 U/L 5. Homogeneous graft perfusion with soft consistency of the parenchyma	No PNF No graft‐related deaths No NAS Follow up: 7 days to 365 days
VAN Leeuwen et al. [31]	The Netherlands	34/54 (63%)	1 DBD‐ECD 33 DCD	Viability testing of initially discarded livers using DHOPE‐COR‐NMP	Liver Assist device	1 to 18: Haemoglobin‐based oxygen carrier 19 to 54 Packed red blood cells	Median CIT: 271 min Median WIT: 29 min Perfusion time: not specified	Within 2.5 h of NMP꞉ Perfusate lactate ≤ 1.7 mmol/L Perfusate pH > 7.35 and < 7.45 bile pH > 7.45 4. In addition, the difference (delta) between bile and perfusate pH, bicarbonate and glucose was determined to assess alkalisation of the bile and glucose reabsorption by the biliary epithelium	No PNF 1 NAS Follow up: 38 months
Quintini et al. [22]	US	15/21 (71%)	4 DBD‐ECD 11 DCD	Viability testing of initially discarded livers	OrganOx metra device	Packed red blood celles	Median CIT: 326 min Median WIT for DCD: 23 min Median perfusion time: 409 min	Within 6 h of NMP: Lowest perfusate lactate level < 4.5 mmol/L or a decrease of 60% from peak in the first 4 h Bile production rate higher than 2 mL/h Stable HA flow of > 0.05 mL/min/g of liver weight and PV flow > 0.4 mL/min/g of liver weight 4. Macroscopic homogenous perfusion and soft consistency	No PNF 1 NAS treated with biliary stent Follow up: 8 weeks to 14 months
Mergental et al. [32]	UK	22/31 (71%)	12 DBD‐ECD 10 DCD	Viability testing of initially discarded livers	OrganOx metra device	Packed red blood cells	Median CIT: 464 min Median WIT for DCD: 21 min Median perfusion time: 587 min	Within 4 h of NMP: Lactate < 2.5 mmol/L and at least 2 of the following criteria: Evidence of bile production pH > 7.30 Metabolism of glucose HA flow > 150 mL/min and PV flow > 500 mL/min Homogenous graft perfusion	No PNF 4 NAS required retransplantation Follow up: 6 months
Cardini et al. [33]	Austria	25/34 (73%)	21 DBD‐ECD 4 DCD	Marginal donor organ quality in 9 patients Logistics in 6 patients Combination of marginal donor quality and logistics in 5 cases Combination of logistics and surgically complex recipients in 2 cases Combination of the marginal organ quality, and logistics as well as surgically complex recipient in 3 cases	OrganOx metra device	Packed red blood cells	Mean CIT: 397 min +/− 144 min Mean WIT for DCD not specified Mean perfusion time: 816 min +/− 386 min	Rapid decrease and maintenance of lactate levels (first 2 h of NMP) Bile output and biliary pH Maintenance of physiological perfusate pH without sodium bicarbonate Exceptionally high OR sharp incline of AST, ALT, LDH	No PNF No NAS Follow up: 20 months
Zhang et al. [10]	CHINA	4/4 (100%)	1 DBD‐ECD 3 DCD	Viability testing of initially discarded livers	Liver Assist device	Leucocyte‐depleted washed red blood cells	Median CIT: 479.5 min Median WIT for DCD: 10 min Median perfusion time: 310 min	Within 4 h of NMP: Perfusate lactate ≤ 2.5 mmol/L Bile production Perfusate pH ≥ 7.30 Stable HA flow > 150 mL/min and PV flow > 500 mL/min	No PNF No NAS Follow up: 6 months
Reiling et al. [34]	Australia	10/10 (100%)	5 DBD‐ECD 5 DCD	Viability testing of initially discarded livers	OrganOx metra device	Packed red blood cells	Median CIT: 307 min Median fWIT for DCD: 17 min Median perfusion time: 738 min	Within 2 h (to 4 h) of NMP: Lactate clearance to < 2 mmol/L Decreasing trend in perfusate glucose concentration by 4 h Physiological pH without the need for sodium bicarbonate Stable HA and PV flows Homogeneous graft perfusion with soft parenchyma consistency Evidence of bile production	No PNF No NAS Follow up: 6 months
Van Leeuwen et al. [35]	The Netherlands	11/16 (69%)	DCD	Viability testing of initially discarded livers using DHOPE‐COR‐NMP	Liver Assist device	Haemoglobin‐based oxygen carriers	Median CIT: 270 min Median WIT for DCD: 32 min Median perfusion time: 598 min	After 2.5 h of NMP: Lactate clearance to ≤ 1.7 mmol/L Perfusate pH 7.35–7.45 Bile production > 10 mL Biliary pH > 7.45	No PNF 1 NAS Median follow up: 12 months
Matton et al. [36]	The Netherlands	Preclinical study: 23 Clinical study: 4/6 (66%)	DCD	Preclinical: 23 declined livers to determine suitable biomarkers of bile duct injuries during NMP Clinical: 6 declined livers for validation of these biomarkers	Liver Assist device	Haemoglobin‐based oxygen carriers	Not specified	After 2.5 h of NMP: Lactate clearance to 1.7 mmol/L Perfusate pH 7.35–7.45 Bile production > 10 mL Biliary pH > 7.48	No PNF No NAS Median follow up: 8.3 months
Ceresa et al. [29]	UK	31/34 (91%)	23 DBD‐ECD 8 DCD	To compare continuous NMP with a post–static cold storage NMP approach	OrganOx metra device	Packed red blood cells	Mean CIT: 361 ± 79 min Median WIT for DCD: 16 min Mean perfusion time: 504 min ± 244 min	Within 4 h: Lactate clearance Glucose metabolism pH maintenance Bile production Perfusate transaminase levels Flow rates	No PNF No NAS Follow up: 12 months
De Vries et al. [36]	The Netherlands	5/7 (71%)	DCD	Viability testing of initially discarded livers using DHOPE‐COR‐NMP	Liver Assist device	Haemoglobin‐based oxygen carriers	Median CIT: 278 min Median WIT for DCD: 27 min Median perfusion time: 493 min	After 2.5 h of NMP: Lactate clearance to ≤ 1.7 mmol/L Perfusate pH 7.35–7.45 Bile production > 10 mL Biliary pH > 7.45	No PNF No NAS Median follow up: 6.5 months
Watson et al. [29]	UK	22/47 (47%)	6 DBD‐ECD 16 DCD	Viability testing of initially discarded livers	OrganOx Metra device	Leucocyte‐depleted washed red blood cells	Median CIT: 383 min Median WIT for DCD: 10 min Perfusion time: not specified	Peak lactate fall ≥ 4.4 mmol/L/kg/h ALT < 6000 iU/L at 2 h Maximum bile pH > 7.5 Bile glucose ≤ 3 mmol/L or 10 mmol less than perfusate glucose Maintain perfusate pH > 7.2 with ≤ 30 mmol bicarbonate supplementation Falling glucose beyond 2 h OR perfusate glucose < 10 mmol/L with subsequent fall during challenge with 2.5 g glucose	1 PNF 4 NAS (3 required retransplantation) Median follow up: 20 months
Watson et al. [28]	UK	12/12 (100%)	3 DBD‐ECD 9 DCD	Viability testing of initially discarded livers	OrganOx Metra device	Packed red blood cells	Median CIT: 427 min Median WIT for DCD: 11 min Median perfusion time: 283,5 min	Lactate clearance Maintaining pH without supplemental bicarbonate Perusate glucose levels Perfusate transaminase levels	1 PNF 3 NAS Median follow up: 20 months
Bral et al. [37]	Canada	9/10 (90%)	5 DBD‐ECD 4 DCD	Safety Assessment of NMP in continuous liver preservation	OrganOx Metra device	Packed red blood cells	Median CIT: 184 min Median WIT for DCD: 23 min Median perfusion time: 690 min	Lactate clearance Perusate pH Perfusate transaminase levels Perfusion vascular stability Hourly bile production	No PNF No NAS Follow up: 6 months
Mergental et al. [10]	UK	5/6 (83%)	1 DBD‐ECD 4 DCD	Viability testing of initially discarded livers	Livers 1 to 5: Liver Assist device The liver 6: OrganOx Metra	Packed red blood cells	Median CIT: 422 min Median WIT for DCD: 31 min Perfusion time: 345 min	Within 3 h of NMP: Lactate clearance to < 2.5 mmol/L or evidence of bile production combined with two of the following criteria: Perfusate pH > 7.30 Hepatic artery flow > 150 mL/min and portal vein flow > 500 mL/min Homogenous perfusion with soft parenchyma consistency	No PNF No NAS No relevant graft‐related complications Median follow up: 7 months

Abbreviations: CIT, cold ischaemia time; DBD‐ECD, extended criteria brain‐dead donors; DCD, donation after circulatory death (none of the DCD livers included in these studies underwent normothermic Regional Perfusion); DHOPE‐COR‐NMP, sequence de Dual hypothermic oxygenated machine perfusion, controlled oxygenated rewarming, and normothermic machine perfusion; NAS, non‐anastomotic biliary strictures; PNF, primary nonfunction; WIT, warm ischaemia time.

In a study conducted by the same research group, a comparative analysis was performed between livers that demonstrated lactate clearance and those that did not. The study revealed that livers in the lactate‐clearing category consistently exhibited a higher probability to sustain a physiological perfusate pH, regulate perfusate haematocrit levels effectively, establish physiological flow rates in both the hepatic artery and portal vein and produce a greater volume of bile compared with their nonlactate‐clearing counterparts. These findings highlight the importance of lactate clearance in differentiating viable livers from nonviable ones [40].

Building on their prior experience, the same team initiated the VITTAL clinical trial, using NMP to evaluate high‐risk livers rejected by all UK centres [23]. The perfusion fluid consisted of PRBC, supplemented with human albumin solution, and the OrganOx Metra device was used. The assessment duration was extended to 4 h. Among the 31 livers evaluated, 22 (12 DBD‐ECD and 10 DCD) met the viability criteria and were transplanted with 100% patient and graft survival at 90 days [32]. However, the 5‐year follow‐up showed graft survival of 72%, with nonanastomotic biliary strictures (NAS) causing graft loss in four cases. Interestingly, lactate clearance was not predictive of ischaemic biliary injury [21].

While lactate clearance is commonly used to evaluate liver viability during NMP, there is no consensus on the optimal threshold values. Different research groups have proposed various cut‐offs. Ghinolfi et al. [41, 42] suggested using a declining lactate trend, regardless of the initial and final lactate levels, as an indicator of liver viability both in old DBD‐ECD and DCD donors with prolonged warm ischaemia time. Reiling et al. [34] proposed a threshold of ≤ 2.0 mmol/L within the first 2 h, whereas the Groningen group suggested a lower threshold of ≤ 1.7 mmol/L [35]. In contrast, Ceresa et al. [43] discarded two livers with lactate 4 mmol/L after 4 h of perfusion, while Cardini et al. [33] deemed livers viable if they reached physiological lactate and pH levels after 2 h. The timing for assessing liver viability based on lactate clearance remains a topic of debate. Hann et al. [44] observed positive outcomes even in livers that cleared lactates slowly, reaching 2.5 mmol/L after 6 h.

In the RESTORE trial [30], 22 declined livers were evaluated using NMP with the OrganOx Metra device. Sixteen livers (11 DBD‐ECD and 5 DCD) met the viability criteria based on a lactate threshold of ≤ 2.2 mmol/L within the first 2 h and all were transplanted without any cases of PNF or graft‐related deaths.

Quintini et al. [22] conducted a study on 21 declined livers perfused with PRBC using the OrganOx Metra device. 15 livers (4 ECD‐DBD and 11 DCD) met the viability criteria and were successfully transplanted. They observed significant variability in lactate levels within the perfusate, which affects the overall lactate load that a liver needs to clear. This variability is due to differences in lactate concentrations in blood‐banked PRBC and a lack of standardisation in the composition and total volume of perfusion solutions. The liver's ability to function as a bioreactor in an isolated perfusion system is also influenced by graft size. To address these issues, the researchers proposed standardised lactic acid clearance (SLAC), which adjusts lactate clearance by graft weight at different time points. Using this approach, they identified two distinct groups of perfused grafts. While all livers cleared lactate within 3 h of starting perfusion, DCD livers showed only 35% clearance at 1 h compared to 60% in DBD‐ECD livers. Interestingly, despite delayed lactate clearance, all livers functioned well after transplantation, and some even showed increased lactic acid concentrations after prolonged preservation without adverse outcomes. Thus, the relevance of SLAC as a viability marker remains uncertain.

Overall, the existing thresholds for lactate clearance and their timing are not well‐established, and there is insufficient evidence to confirm whether transplanting livers with higher lactate levels leads to complications such as PNF. For example, Nasralla et al. [45] documented a case of PNF despite acceptable lactate clearance during NMP, while Watson et al. [29] observed that the only liver that experienced PNF showed a perfusate lactate level of 2.5 mmol/L after 90 min of perfusion. In their Phase 1 trial, Liu et al. [46] successfully transplanted three livers that did not clear lactate to normal levels—livers that would not have been transplanted based on criteria established by other groups [29, 35, 37, 40, 41]. Thus, while lactate levels remain an important marker in organ acceptance, their predictive value during NMP is still unclear.

### 2.3 Bile Production

Bile production is a crucial function of the liver, primarily involving hepatocytes and cholangiocytes that line the bile ductules. This process is meticulously regulated through a complex interplay between secretion and absorption mechanisms. The primary driving force behind bile formation is osmotic filtration, which depends on the active transport of bile acids and other solutes. This process requires a continuous supply of energy in the form of adenosine triphosphate (ATP) [47]. In LT, many clinical studies on machine perfusion have used bile production as a criterion for assessing viability [18, 30, 33, 43, 44, 45, 48] (Table 2). While specific cut‐off values for bile production are often not specified, the Groningen group, based on their studies combining dual hypothermic oxygenated machine perfusion (DHOPE), controlled oxygenated rewarming (COR) and NMP (DHOPE‐COR‐NMP) sequence, set a threshold of > 10 mL of bile production within 2.5 h of NMP [33, 40] (Figure 1B). However, recent studies suggested that the predictive value of bile production in evaluating liver function may be overstated, as graft loss has been reported despite proper bile production [28, 29].

For example, Ceresa et al. [43] identified four livers with insufficient bile flow within 4 h of NMP, all of them were transplanted, displaying immediate function without relevant graft quality‐related complications. Similarly, Zhang et al. [49] reported that four transplanted livers, which would have been declined based on the Groningen criteria (having produced less than 10 mL of bile within 2.5 h), demonstrated immediate full function. It is important to acknowledge that bile production primarily reflects hepatocyte viability rather than cholangiocyte function [50]. This is supported by studies showing that bile volume does not consistently correlate with the risk of post‐transplant cholangiopathy [28, 29, 35]. For example, in a clinical viability study involving 12 discarded donor livers assessed using NMP and subsequently transplanted, two livers with high bile production exhibited signs of ischaemic cholangiopathy two months after LT [35]. Similarly, the VITTAL study reported that although transplanted livers met hepatocyte viability criteria, including bile production, 45% of the recipients developed bile duct irregularities as seen on magnetic resonance cholangiography (MRC). Among the 10 DCD livers, three (30%) developed NAS requiring retransplantation [21]. Finally, the complete absence of bile flow during NMP could sometimes be attributed to technical issues, such as the misplacement of the biliary drain or leakage along the drain [35]. This observation suggests that the absence of bile production during NMP may not necessarily indicate nonviability, as successful transplantations have been reported even when bile production was absent [45].

### 2.4 Glucose Metabolism

The liver plays a pivotal role in the regulation of glucose homeostasis through various metabolism pathways, including glycogenesis, glycolysis and gluconeogenesis [51]. In response to ischaemia, cells adapt to anaerobic metabolism for energy, leading to decreased ATP levels and increased glycogenolysis, resulting in higher glucose levels in the perfusate during NMP [18, 36].

In viable livers, elevated glucose concentrations in the perfusate inhibit glycogenolysis and stimulate glycogen synthesis, leading to a reduction in perfusate glucose levels [29] (Figure 1C). However, the absence of increased perfusate glucose levels may indicate depletion of glycogen stores and significant damage to hepatocytes [18]. To rule out severe hepatocyte injury, glucose challenge tests are recommended.

Adding glucose to the perfusate triggers gluconeogenesis, which is active only in cells capable of performing aerobic metabolism. If the proportion of healthy hepatocytes in the liver is significant, a decrease in the glucose levels of the perfusate can be observed [25, 26, 27, 32].

Viable livers are also known to metabolise glucose in response to insulin. A recent study by Eshmuminov et al. [25] indicated that glucose supplementation is essential to maintain normal perfusate glucose levels over seven days of ex situ perfusion, especially in livers that were later considered unsuitable for transplantation. Functionally viable livers initiate glucose release through gluconeogenesis, following insulin administration. This could be an interesting factor for assessing liver metabolic health in future studies.

### 2.5 Perfusate pH

The liver is an important regulator of acid–base balance and achieves homeostasis mainly through lactate metabolism, albumin synthesis, ketogenesis and urea production [33, 36]. During NMP, reduced pH in the perfusate is often observed, especially when the donor liver has sustained significant damage or undergone prolonged ischaemia, leading to tissue hypoxia and a shift to anaerobic metabolism. The standard practice, as suggested in several studies, is to maintain pH within a normal physiological range, ideally between 7.3 and 7.45 (Figure 1D) [10, 26, 47, 52]. Bicarbonate is regularly administered, both initially and throughout NMP, depending on the pH of the perfusate [33]. The liver's ability to regulate perfusate pH has been recognised by several groups as a crucial viability marker, with healthy livers expected to develop an alkaline perfusate over time [40, 41, 45]. However, pH balance is influenced by multiple factors including perfusate composition, exogenous bicarbonate supplementation, partial pressure of carbon dioxide and lactate levels. Consequently, a precise assessment of liver viability requires careful consideration of all these factors. The amount of exogenous bicarbonate required to maintain a pH above 7.3 can serve as an indicator of liver health. In the study by Watson et al. [29], among 47 livers initially deemed unsuitable for transplantation and assessed with NMP, 22 (6 ECD‐DBD, 16 DCD) met the viability criteria and were transplanted. Three livers required more than 30 mmol bicarbonate, one of which was transplanted and underwent PNF. Consequently, the authors proposed a maximum bicarbonate addition of 30 mmol/L to maintain a perfusate pH above 7.2 as part of their viability criteria.

However, in the study by Quintini et al. [22], four transplanted livers failed to develop an alkaline pH, and two livers required a large amount of bicarbonate infusion (> 40 mmol bicarbonate). Despite this, all these livers were transplanted and functioned well, suggesting that pH requires needs further investigation before being considered a reliable viability marker.

### 2.6 Production of Coagulation Factors

Liver synthesis produces most coagulation factors. Specifically, hepatocytes generate factors such as FV, FVII, FIX, FX and antithrombin, while hepatic sinusoidal cells are responsible for FVIII [53, 54]. Despite the use of heparin as an anticoagulant during NMP, coagulation factor production remains a marker for liver viability. Eshmuminov et al. [25] demonstrated that factor V levels were higher in well‐functioning livers than in nonfunctioning ones after 48 h of perfusion, though this difference diminished over time. Additionally, it has been observed that a reduction in the international normalised ratio (INR) during machine perfusion may reflect liver recovery and the resumption of coagulation factor production [55, 56, 57].

Poor‐quality livers often show greater fibrinolysis and are more vulnerable to IRI [58]. Studies have shown that NMP performed at the end‐ischaemia phase can trigger fibrinolysis, and elevated D‐dimer levels in the perfusate may indicate severe ischaemic damage and poor liver viability [58].

While markers such as INR and fibrinolysis can provide insights into liver viability, the use of some coagulation factors as markers is limited due to the interference of heparin, which is routinely used in perfusion protocols. Moreover, the increase in fibrinogen, FV and FIX levels does not consistently correlate with peak AST levels post‐transplantation. Although the accumulation of anticoagulant factors is common, their concentrations are often lower in severely damaged livers [53]. Therefore, while coagulation factors offer potential as viability markers, their predictive accuracy remains uncertain, and defining clear thresholds remains a challenge.

### 2.7 Hemodynamic Stability

Under physiological conditions, hepatic artery flow is regulated by the autonomic nervous system and vasoactive substances, which depend on liver signals. In contrast, portal vein flow functions independently [59]. During NMP, the flow in both the arterial and portal systems is influenced by vascular resistance and perfusion pressure [25]. Prolonged ischaemia can impair hepatic microcirculation, leading to increased vascular pressure and compromised liver function [60]. This deterioration is typically caused by a reduction in vasoprotective mechanisms and endothelial cell dysfunction. Livers with significant macrosteatosis are more prone to severe sinusoidal lining cells deterioration, leading to decreased perfusion flow [61]. Increased vascular resistance during machine perfusion often indicates suboptimal liver function [62]. As a result, many clinical studies on NMP have established specific blood flow thresholds, greater than 150 mL/min in the hepatic artery and greater than 500 mL/min in the portal vein (Figure 1E,F), as critical indicators of liver viability [10, 30, 32]. Several case studies have reported decreased flow rates towards the end of prolonged NMP, indicating sinusoidal exhaustion and severe damage [25, 28]. In previous cases in which there was compromised flow and elevated resistance, significant injury was obvious upon histological examination. Therefore, indicators of perfusion quality should be considered late‐stage markers of organ dysfunction, which explains why most studies have been unable to distinguish between viable and nonviable livers based solely on perfusion flow assessments [27, 29, 52]. Alternatively, assessing the reaction of the liver to vasoactive medications such as epinephrine can be informative. In their study, Eshmuminov et al. [25] demonstrated that livers that did not respond to such medications despite high vasoconstrictor infusion, a condition known as vasoplegia, were often associated with histological signs of structural deterioration and elevated levels of markers indicating injury and inflammation (such as alanine aminotransferase [ALT] aspartate aminotransferase (AST), interleukin 6, interleukin 10 and uric acid), in contrast to those that did respond to vasoactive drugs.

### 2.8 Liver Transaminases

Ischaemic liver injury primarily damages hepatocytes and sinusoidal endothelial cells, causing changes in cell membrane permeability, which may lead to leakage of transaminases into the perfusate [63, 64].

Transaminases, specifically ALT and AST, mainly located in the cytoplasm of various tissues including the liver, are commonly used to assess liver injury during NMP. While AST and ALT release has been predictive of recipient survival in a large animal model [50], their predictive value for post‐transplantation outcomes remains limited [10, 65]. Several factors contribute to this limitation, including the ‘wash‐out’ phenomenon and liver size, both of which can affect transaminase levels during perfusion [66]. The ‘wash‐out’ effect, influenced by the volume of preservation fluid, may result in artificially low transaminase levels. To address these challenges, Quintini et al. [22] proposed a normalisation method that considers the liver weight and the volume of preservation fluid when evaluating transaminase levels. It is also important to note that hemolysis can affect AST levels in the perfusate, while ALT, which is more specific to the liver, offers greater accuracy for assessing liver function.

In clinical practice, there is no universally defined cut‐off values for perfusate transaminase levels. Grafts with significantly elevated transaminase levels in the perfusate, often exceeding 9000 IU/L, are typically considered for rejection, although this depends on individual centres' experience and preferences. For instance, Watson et al. [29] reported a case of PNF in a patient who received a liver with a perfusate ALT level of 9490 IU/L. Similary, Van Leeuwen et al. [35] demonstrated that livers rejected for transplantation due to failing other viability tests exhibited perfusate ALT levels > 6000 IU/L (Figure 1G).

In addition, the pattern of transaminase concentration during perfusion can provide valuable insights. Stable transaminase levels suggest an absence of ongoing hepatocellular injury, while a sharp increase in transaminase levels may indicate severe liver injury and serve as an indirect marker of poor outcomes [29, 33]. Recent studies by Nasralla et al. [45], published in 2018, explored the relationship between baseline perfusate ALT levels and post‐transplant outcomes, indicating that higher baseline ALT levels may be associated with poorer results. In contrast, Ghinolfi et al. [41] found no significant association between peak perfusate AST levels and post‐transplant transaminase levels.

These findings highlight the ongoing challenges of using transaminases as reliable markers for assessing liver function during perfusion. Therefore, it is important to interpret them carefully, considering factors such as liver weight, the volume of perfusion fluid used and the potential impact of hemolysis.

### 2.9 Cholangiocellular Viability Criteria

Most liver viability studies have focussed on the assessment of hepatocellular injury and function. However, the risk of post‐transplant graft failure is not solely determined by the extent of the hepatocellular injury. Biliary injury also plays a significant role. In particular, DCD livers are more susceptible to develop biliary complications, notably NAS, which can lead to graft loss [24, 63, 67, 68].

It has been observed that livers with minimal hepatocellular damage can still exhibit severe biliary duct injury (BDI), which might go unnoticed without specific biliary viability criteria [69]. In a clinical study of 12 initially declined liver grafts later deemed suitable for transplantation during NMP, 25% developed post‐transplant cholangiopathy despite having demonstrated adequate hepatocellular function during NMP [28]. The VITTAL study further revealed that although the transplanted livers met hepatocellular viability criteria, including bile production, 45% of patients showed biliary irregularities on MRC, and 18% required retransplantation due to biliary strictures [21, 32].

This difference in hepatocellular and cholangiocellular injuries is attributed to the higher sensitivity of cholangiocytes to IRI and their slower postischaemic recovery of intracellular ATP compared with hepatocytes [4, 69, 70].

Two key functions of a healthy biliary epithelium are active bicarbonate secretion and glucose reabsorption, resulting in alkaline biliary pH and low glucose concentrations in the bile [71, 72]. Biliary epithelial cells from livers with high BDI are unable to secrete sufficient bicarbonate to raise biliary pH [69]. Maintaining an alkaline biliary pH is crucial in protecting biliary epithelial cells from the toxic effects of hydrophobic bile salts, known as the ‘biliary bicarbonate umbrella’ [69, 73]. Thus, low biliary pH and bicarbonate levels not only indicate biliary injury/dysfunction but also may contribute to further biliary injury due to the absence of this protective mechanism [5].

The glucose concentration in the bile could also be of interest, although interpreting glucose values in the bile is complex. Postischaemic reperfusion of a liver graft generally leads to an increase in glucose levels in the perfusion fluid due to glycogenolysis [31], which influences the concentration of glucose in the primary bile produced by hepatocytes. High biliary glucose concentrations can impair glucose reabsorption, even when the biliary epithelium is intact. Therefore, biliary glucose levels should be interpreted relative to glucose levels in the perfusion fluid [69, 72].

Given these findings, various preclinical and clinical studies have suggested biliary pH, bicarbonate and glucose levels as potential criteria for cholangiocellular viability during NMP.

In their original study involving 12 livers (3 ECD‐DBD, 9 DCD), the Cambridge team measured the biliary pH during NMP. While these measurements were not used for decision‐making purposes, researchers were pioneers in proposing bile pH as a crucial viability criterion. They noted a high incidence of cholangiopathy, which was associated with the inability of the liver to produce an alkaline pH during NMP [28].

In 2018, Watson et al. [29] observed that livers unable to produce bile with a pH greater than 7.4 during NMP developed postoperative cholangiopathy (Figure 1H). Livers achieving a biliary pH > 7.5 showed minimal stromal necrosis in major intrahepatic ducts, while those with a bile pH < 7.5 exhibited moderate or severe stromal necrosis.

In 2019, Matton et al. [69] conducted a study on 23 human donor livers, applying a DHOPE‐COR‐NMP sequence, to identify biomarkers of graft viability. Their research, which included a preclinical phase with 23 declined livers and a clinical validation phase with six livers, revealed that high biliary bicarbonate levels (> 18 mmol/L), biliary pH (> 7.48), low biliary glucose (< 16 mmol/L), a bile/perfusate glucose ratio (< 0.67) and low biliary lactate dehydrogenase (LDH) levels (< 3.7 U/L) were reliable indicators for predicting reduced post‐transplant biliary complications.

The Groningen group conducted a prospective clinical trial known as the DHOPE‐COR‐NMP trial [35], where nationwide declined livers were subjected to NMP for viability assessment, preceded by 1 h of DHOPE as a resuscitation phase and 1 h of COR, using a perfusion fluid containing a haemoglobin‐based oxygen carrier. During the first 2.5 h of NMP, hepatobiliary viability was assessed using predefined criteria, including perfusate lactate levels below 2.5 mmol/L, bile production over 10 mL and bile pH greater than 7.45. Livers meeting these criteria were considered viable and were accepted for transplantation. Of 16 perfused livers (DCD), 11 (69%) met the viability criteria and were transplanted. The median follow‐up for the patients who received a DHOPE‐COR‐NMP liver was 12 months. The trial demonstrated actuarial graft survival rates of 100% at 3, 6 and 12 months. However, one recipient developed cholangiopathy 4 months after transplantation, requiring a biliary anastomosis constructed via Roux‐en‐Y hepatico‐jejunostomy.

The same Groningen team has recently reported its results using the DHOPE‐COR‐NMP sequence [74]. One hundred and five procedures were performed on initially discarded livers to test viability using the previously defined markers, and 69 livers were deemed transplantable and transplanted (rescue rate 66%). The majority of livers, 98%, were from DCD donors. The median functional Donor Warm Ischaemia Time (DWIT) (start defined as SpO_2_ less than 80% or mean arterial pressure less than 60 mmHg) (ref:) was 30 min. Of the 69 livers that were successfully transplanted after sequential DHOPE‐NMP, 31 livers had a functional DWIT ≥ 30 min. In univariate logistic regression analysis, neither functional DWIT nor asystolic warm ischaemia time were associated with liver discard after NMP assessment. However, cold ischaemia time emerged as a strong predictor and remained the only significant risk factor for poor graft viability in multivariate analysis. The graft and recipient survival rates at 1 and 3 years were 93% and 91% and 99% and 97%, respectively. Two patients (3%) developed NAS and required retransplantation. Based on these findings, the viability criteria were modified using the delta between bile and perfusate levels of pH, bicarbonate and glucose, rather than their absolute biliary values (Table 1) (Figure 1I–K). No cases of NAS were observed using these cholangiocellular viability criteria [64].

The results of the VITTAL study, which employed end‐ischaemic NMP, indicated that this approach exposes the graft to ischaemia–reperfusion injury, to which the bile ducts are extremely sensitive [75]. Since the beginning of the VITTAL trial in 2016, the Cambridge team shared a similar experience showing high percentages of biliary complications after NMP of livers from DCD donors [28, 29]. It has been demonstrated that a short period of HOPE reduces ischaemia–reperfusion injuries [76, 77]. The Groningen group had shown a beneficial effect of the combination of arterial and portal hypothermic perfusion (DHOPE), COR and NMP (DHOPE‐COR‐NMP) on cholangiocellular function. Starting with a short period of DHOPE before NMP, along with the use of cholangiocellular viability criteria during NMP, could help reduce post‐transplant morbidity and the risk of graft loss due to NAS. It should be highlighted that viability assessment criteria used in the DHOPE‐COR‐NMP sequence might not be directly applicable to NMP protocols. Additionally, the use of artificial hemogloblin‐based oxygen carrier is generally prohibited in the most countries.

Recent studies have introduced new biomarkers, including microRNAs, as well as markers of tissue integrity and regeneration. In a study conducted by Verhoeven et al. in 2013 [78], microRNA expression in the graft preservation solution of 20 grafts that developed NAS was compared with 37 that did not. These findings revealed that the ratio of hepatocyte‐derived microRNAs to cholangiocyte‐derived microRNAs was higher in grafts that later developed NAS. More recently, Matton et al. [79] analysed miRNA levels in both the perfusate and bile during NMP of 12 human liver grafts that were initially declined. They found a significant correlation between miRNAs originating from cholangiocytes and the extent of biliary damage and functionality, as evidenced by the levels of LDH, bilirubin and bicarbonate.

In 2018, Liu et al. [80] focussed on the regenerative capacity of the biliary systems over a 24‐h NMP period in 10 rejected livers. Their findings highlighted the regeneration of cholangiocytes and peri‐biliary glands, as indicated by the increased Ki‐67 staining in bile duct biopsies.

The Groningen group conducted a proteomic analysis of bile samples collected during the DHOPE‐COR‐NMP sequence from 55 human donor livers. Their longitudinal study during NMP identified proteins indicative of early cellular damage, followed by increased expression of proteins associated with secretory activities and immune responses. Livers deemed suitable for transplantation based on bile chemistry showed protein profiles suggestive of regenerative activities, including cell proliferation, in contrast to those with suboptimal bile chemistry. This observation was further supported by the presence of gene transcripts related to liver tissue regeneration before machine perfusion. These comprehensive proteomic and transcriptomic analyses of bile and the liver offer insights into the molecular processes that occur during NMP and help refine the criteria for assessing liver viability.

## 3 Conclusion and Perspectives

Assessing the viability of liver grafts represents a significant challenge, primarily due to the absence of a universally recognised definition of viability. In recent years, various parameters have been proposed to determine graft viability during machine perfusion. However, a definitive and widely accepted indicator for confirming a liver's suitability for transplantation has yet to be established. Practices vary considerably among research groups, each employing their own perfusion techniques and evaluation criteria. Most markers were defined in the livers that declined for transplantation and the cut‐off values of these biomarkers have been applied in clinical trials with promising results.

However, it remains uncertain whether a liver that does not meet these criteria could still function adequately if transplanted. Clinicians should adopt a combined approach using both hepatocellular and cholangiocellular viability criteria, such as those that assess lactate clearance and bile quality, to better predict post‐transplant outcomes.

A more effective strategy might involve developing robust graft risk scores that integrate both perfusion parameters and biomarkers, offering a more nuanced correlation with long‐term graft survival compared with the current binary classification of grafts as either viable or nonviable. Rare events such as PNF or ischaemic cholangiopathy, while valuable for establishing parameter thresholds, require large patient cohorts for significant research. Comprehensive randomised clinical trials are essential to validate the reliability of these viability criteria. The practical use of potential biomarkers, such as microRNAs, ATP production, metabolic profiling or multiomics, is often limited by the lengthy processing times required, which could delay transplantation decisions. Therefore, real‐time assessment of biomarkers is crucial for making timely decisions about organ acceptance for transplantation.

## Author Contributions

**Heithem Jeddou:** conceptualisation, methodology, writing – original draft preparation, writing – review and editing. **Stylianos Tzedakis:** writing – review and editing, validation. **Mohamed Ali Chaouch:** methodology, validation. **Laurent Sulpice:** supervision, validation. **Michel Samson:** supervision, validation. **Karim Boudjema:** supervision, validation.

## Conflicts of Interest

The authors declare no conflicts of interest.

## Data Availability

The authors have nothing to report.
